# Efficacy of Octocog Alfa (Advate) in a Child with Type 3 von Willebrand Disease and Alloantibodies

**DOI:** 10.3390/jcm6090085

**Published:** 2017-09-18

**Authors:** Gianluca Sottilotta, Francesca Luise, Elisabetta Massara, Vincenzo Oriana, Angela Piromalli

**Affiliations:** UOSD Microcitemie–Emostasi e Trombosi, Grande Ospedale Metropolitano “Bianchi-Melacrino Morelli” Reggio Calabria, Reggio Calabria 89100, Italy; francescaluise@alice.it (F.L.); elisabetta.massara@gmail.com (E.M.); voriana@libero.it (V.O.); a.piromalli@alice.it (A.P.)

## 1. Introduction

Von Willebrand disease (VWD) is the most frequent inherited bleeding disorder and is caused by either a quantitative and/or qualitative defect of the multimeric glycoprotein von Willebrand factor (VWF). The revised classification identifies VWD Type 1 and Type 3 characterized by quantitative VWF defects, and the VWD Type 2 characterized by qualitative VWF defects [1]. Approximately 70% of patients have Type 1, usually associated with a mild bleeding history characterized by prolonged oozing after minor and major surgery and by mucosal tract hemorrhages such as epistaxis and menorrhagia. Twenty to twenty-five percent of patients have Type 2, characterized by frequent bleeding episodes in the mucosal tract and rarely soft tissue hemorrhages because patients have relatively high levels of Factor VIII (FVIII). VWD Type 3 involves a virtually complete quantitative VWF deficiency. Its inheritance is autosomal recessive, and is the result of the inheritance of two null alleles. The prevalence of Type 3 is relatively low, with 0.5–1 cases per million in the general population [2]. Patients with VWD Type 3—the most severe form—are characterized by a severe quantitative defect and very low levels of both VWF and FVIII, and are clinically characterized by excessive mucocutaneous bleeding as well as musculoskeletal bleeding such as muscle hematomas and hemarthroses [3]. In VWD, the aim of therapy is to correct the dual defect of haemostasis; i.e., the abnormal platelet adhesion—aggregation and the abnormal intrinsic coagulation due to low FVIII levels. The mainstays of treatment are autologous replacement therapy with desmopressin and allogenic replacement therapy with VWF/FVIII or VWF concentrates devoid of FVIII. Desmopressin is not an effective treatment for all VWD patients. There are also adjuvant therapies with antifibrinolytic amino acids such as tranexamic acid and epsilon aminocaproic acid, which improve haemostasis without affecting plasma VWF levels [4]. Anti-VWF alloantibodies develop after multiple infusions in 10% to 15% of patients with Type 3 VWD, in whom subsequent treatment with VWF concentrates is not only ineffective, but may cause post-infusion severe allergic reactions like abdominal pain, lumbar pain, hypotension, and anaphylactic shock, because of the formation of immune complexes that activate the complement system. These reactions may be life-threatening [5]. In contrast to FVIII antibodies, because assays for anti-VWF antibodies are not standardized, they can be difficult to measure. Treatment of patients with anti-VWF alloantibodies can be quite challenging, as experience in managing these patients is limited. Good hemostasis has been reported with the use of recombinant FVIII (rFVIII) [6]: despite the shortened half-life of FVIII in the absence of its stabilizer, VWF, high-dose infusions of rFVIII can maintain hemostatic levels of FVIII. Successful immune tolerance induction (ITI) similar to that seen in patients with inhibitors of FVIII has been reported; however, because of the lack of experience in VWD, whether or not ITI is safe or effective in all patients with anti-VWF antibodies remains to be seen [7]. Bypassing agents such as recombinant factor VIIa (rFVIIa) has been successful with either bolus dosing or continuous infusion [8]. Platelet concentrates can be considered as additional treatment options [9].

## 2. Case Presentation

We report the case of an 18-month-old boy affected by VWD Type 3 who developed anti-VWF alloantibodies.

Frequent bruising of the skin appeared since birth. At the age of two months, because of a left facial hematoma, he was subjected to radiological investigations that led to the diagnosis of a cystic ameloblastoma—a benign odontogenic tumor present in his left jaw bone. The diagnosis of ameloblastoma and the following surgical intervention were performed in another hospital before the diagnosis of VWD, because of the difficulty in having a quick assay of the coagulation factors whose deficiency could explain the prolonged activated partial thromboplastin time (aPTT) evidenced in the pre-operative blood testing. Fresh frozen plasma (FFP) was infused before the surgery, with a partial success in terms of anti-hemorrhagic efficacy because of a temporary bleeding from the surgical wound after two days. The diagnosis of VWD Type 3 was performed after surgery; the results evidenced FVIII: 3.5%, VWF Antigen (VWF:Ag): 2%, VWF Ristocetin Cofactor (VWF:RCo): 10%. An on-demand treatment with Talate (Human Coagulation Factor VIII, Human Von Willebrand Factor, Baxalta) was prescribed, but during the following months, the child was only treated with Ugurol (Tranexamic Acid, Rottapharm) 25 mL/kg administered orally when necessary, because of the absence of significant clinical bleedings. 

After several months, the child was hospitalized due to a palate bleeding after an injury; infusions of Talate (100 UI/kg) every 24 h were administered until hemorrhage stopped, then the patient was discharged with a prescription of Ugurol three times per day orally. After a week, the child was treated with Talate 100 UI/kg at home for a prolonged oral bleeding due a dental eruption: at the end of the infusion, onset of pallor, cyanosis, weakness, tremor, and hyperpyrexia occurred. These symptoms lasted about 10 min, and then disappeared. The following day after symptom onset, the patient underwent blood tests at our centre for dosing factors and anti-VWF alloantibodies. The exams performed 12 h after the infusion revealed the presence of anti-VWF antibodies: Anti VWF:Ag: 15.6 Bethesda Units (B.U.), Anti VWF:RCo 30.4 B.U., and low levels of FVIII and VWF (FVIII: 5.7%, VWF:Ag: 0%, VWF:RCo: 11%). A week later, the patient had a gingival bleeding: Novoseven (Activated Eptacog Alfa, Novonordisk) 100 mcg/kg every 2 h was started at home, but because the first infusion of Novoseven was not effective the child was also hospitalized because of severe bleeding. Blood tests showed a low hemoglobin (Hb) level (6 g/dL); the child was treated with red blood cell (RBC) transfusions, washed to remove the traces of VWF in order to avoid an anamnestic response; Novoseven 100 mcg/kg every two hours was continued for 24 h, but symptoms worsened with the onset of hematemesis and melena: because Hb level was 4.5 g/dL, additional RBC transfusions were administered. The therapy with Novoseven was stopped due to inefficacy and replaced with Octocog Alfa (Advate, Shire), a third-generation plasma/albumin-free rFVIII. It was started with a bolus of 100 IU/kg followed by continuous infusion at 50 IU/h. At the same time, intravenous omeprazole as gastroprotective and Ugurol by continuous infusion at 4 mL/h were started.

The first bolus of rFVIII stopped the gingival bleeding temporarily, but it started again as slow oozing after about two hours and continued throughout the first day of continuous infusion with Advate. The observation of short interruption of bleeding following the Octacog bolus led to the decision to change the mode of administration: we suspended the continuous infusion and started the administration of 100 IU/kg boluses every 6 h. After this therapeutic change, the bleeding stopped definitely. After two days without hemorrhages, the frequency of the boluses was reduced to every 8 h for the first day, then to every 12 h for another day, then it was discontinued. The day after Advate discontinuation, a coagulation assay was performed: results were FVIII: 3.5% VWF:Ag: 0%, VWF:RCo: 11%, Anti VWF:Ag: 3 B.U., Anti VWF:RCo: 5.4 B.U. The infusion of Ugurol was continued without any changes until complete discontinuation of Advate suspension, and then it was administered orally at 25 mg/kg every 8 h: the same therapy was prescribed after hospital discharge for the prevention of further gingival bleedings due to dental eruptions.

## 3. Discussion

Alloantibodies against VWF may develop in approximately 10% of patients suffering from VWD Type 3 who underwent multiple transfusions and especially in carriers of large deletions of the VWF gene. Because of severe anaphylactic life-threatening reactions, the use of concentrates rich in VWF is contraindicated in these patients [10]. In literature, clinical experience on the therapeutic management of this disease is limited [11]: some authors have described the clinical efficacy of rFVIII concentrates, and they also reported an allergic reaction after a first-generation rFVIII bolus, probably due to the presence of VWF traces. Novoseven has been used successfully in the management of bleeding [12], as intermittent bolus or as continuous infusion, at the same doses used in patients with severe haemophilia A and anti-FVIII inhibitor; in our case, however, the treatment with recombinant activated factor VII did not prove effective. Neither did the continuous infusion of rFVIII demonstrate a persistent anti-hemorrhagic action, probably because the FVIII levels were not sufficient to maintain the minimum haemostatic threshold.

Therefore, after the observation of the temporary interruption of the gingival bleeding, due to the initial bolus of rFVIII, we decided to treat the patient with boluses of rFVIII only; this choice has proven effective and lasting. 

## 4. Conclusions

Our experience shows the efficacy of Advate in the on-demand treatment of VWD Type 3 and inhibitor. The effectiveness of the rFVIII in our case is based on the temporary increase in FVIII levels. The difficult patient’s venous access due to the age did not allow us to test the FVIII levels after each rFVIII infusion, which would be useful in determining the frequency of administration. To date, there are no retrospective or prospective studies on the treatment of patients with VWD Type 3 and inhibitor: available data is derived from individual experiences described in few case reports only. Appropriate registers can contribute in the future to further improve the knowledge on this VWD complication and to establish appropriate treatment guidelines.
